# Spermatic cord liposarcoma mimicking recurrent incarcerated inguinal hernia: A case report

**DOI:** 10.1016/j.eucr.2026.103517

**Published:** 2026-06-23

**Authors:** Murilo Augusto Ferreira, Gabriel Andrade Agareno, Fernando Aparecido Pazini, Patrícia Mendes dos Santos Angolini, Mateus Santos Matos, Viktor Dias Magalhães, Manuela Mascaro Dias, Daniel Salerno Muzilli, Thiago da Silveira Antoniassi

**Affiliations:** Faculdade de Medicina de São José do Rio Preto (FAMERP), Brazil

**Keywords:** Liposarcoma, Spermatic cord, Testicular neoplasm, Hernia, Inguinal

## Abstract

Spermatic cord liposarcoma is a rare malignant neoplasm often misdiagnosed as benign conditions. This similarity may lead to inadequate surgical approaches.

A 55-year-old man with a prior history of inguinal hernioplasty presented with an incarcerated scrotal mass. The initial hypothesis of recurrent hernia was ruled out after negative surgical exploration. Imaging suggested a lipomatous lesion, and a resection with left orchiectomy was performed. Histopathological examination confirmed liposarcoma.

Spermatic cord liposarcoma is a differential diagnosis of inguinoscrotal masses. Radical resection with negative margins is the gold standard treatment. Long-term surveillance is mandatory due to the risk of recurrence.

## 1 Introduction

Paratesticular tumors represent an heterogeneous and rare group of neoplasms arising from the structures of the spermatic cord, epididymis, testicular tunics, and vestigial appendages. Approximately 70% to 80% of these tumors are benign and lipoma is the most common. Among malignant tumors, spermatic cord liposarcoma (LPS) is the predominant histological subtype in adults, accounting for approximately 30% of sarcomas in this region.

The usual clinical presentation is a slow-growing and generally painless inguinoscrotal mass, which makes it clinically indistinguishable from much more prevalent conditions such as inguinal hernias, hydroceles, spermatoceles, or chronic epididymitis. This scenario represents a significant diagnostic challenge and may lead to inadequate surgical approaches and delay in definitive treatment.

We report the case of a patient with spermatic cord liposarcoma whose initial presentation mimicked a recurrent incarcerated inguinal hernia.

## 2 Case presentation

A 55-year-old male patient with hypertension and a history of left inguinal hernioplasty one year prior presented to the emergency department reporting an increase of the left scrotum over the past five months, associated with intermittent pain triggered by exertion, with worsening of pain over the previous two days. He denied fever or changes in urinary or intestinal systems. No relevant family history was reported.

On physical examination a significant increase in the volume of the left hemiscrotum was observed, without local inflammatory signs. The palpable mass was irreducible and had fibroelastic consistency. The ipsilateral testis was difficult to distinguish.

Initially evaluated by the General Surgery group, the diagnostic hypothesis of recurrent incarcerated left inguinal hernia was established and urgent surgical exploration was suggested.

An exploratory inguinotomy was performed, identifying the polypropylene mesh from the previous hernioplasty, intact and well positioned, with no evidence of a hernia sac or abdominal wall defect. A diagnostic laparotomic exploration by an infraumbilical approach was performed during the same procedure, which also showed no evidence of any abdominal wall hernia.

Given the absence of a diagnosis, a scrotal ultrasound was requested. It demonstrated a “heterogeneous expansive lesion, predominantly echogenic, with interspersed hypoechoic areas, measuring 11.3 × 11.0 × 10.2 cm (volume of 662 mL), located in the posterior wall of the scrotum, with no vascular flow on Doppler.” The radiologist suggested the possibility of a lipomatous lesion (Fig. 1).

**Fig. 1:**
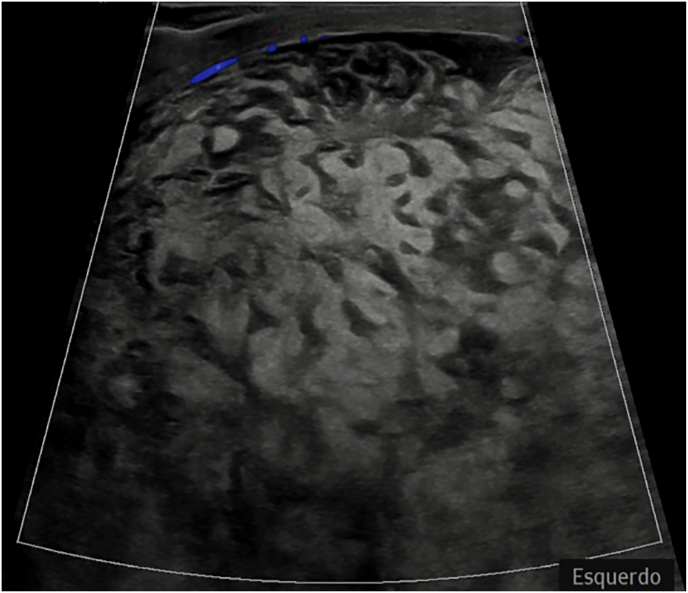
Scrotal ultrasonography. Heterogeneous expansile lesion located in the posterior wall of the left hemiscrotum.

For better investigation, a pelvic Magnetic Resonance Imaging (MRI) was performed and described an “increased volume of the left testicular sac (1200 ml) due to a predominantly cystic collection-like image, with well-defined walls, no contrast enhancement, with an approximate volume of 800 ml, associated with a large amount of fatty tissue.” (Fig. 2)

**Fig. 2:**
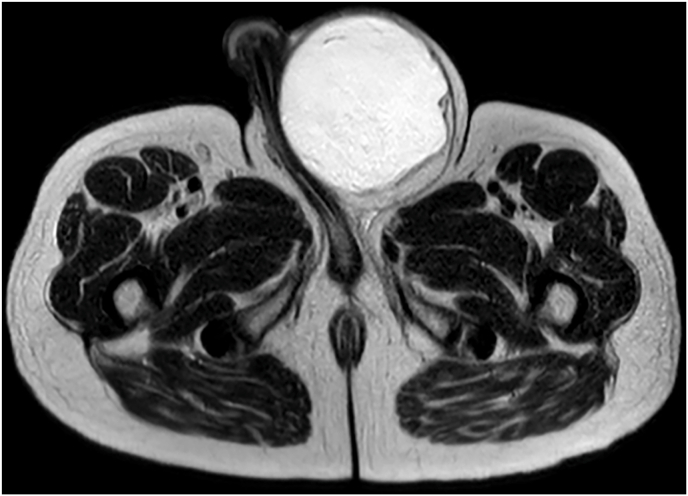
T2-weighted pelvic magnetic resonance imaging. Well-defined predominantly cystic collection measuring approximately 800 cm^3^ associated with extensive adipose tissue within the left hemiscrotum.

Based on the imaging findings, the patient was referred to the Urology group.

A surgical exploration was then performed with a scrotal approach, through an incision in the median raphe. Intraoperatively, a large lipomatous mass (yellowish and multilobulated) was identified in the left hemiscrotum, extratesticular, but closely adherent to the testis and the elements of the spermatic cord (Fig. 3). Complete en bloc resection of the mass was performed. Dissection did not allow preservation of the testicular vasculature, requiring a left radical orchiectomy.

**Fig. 3:**
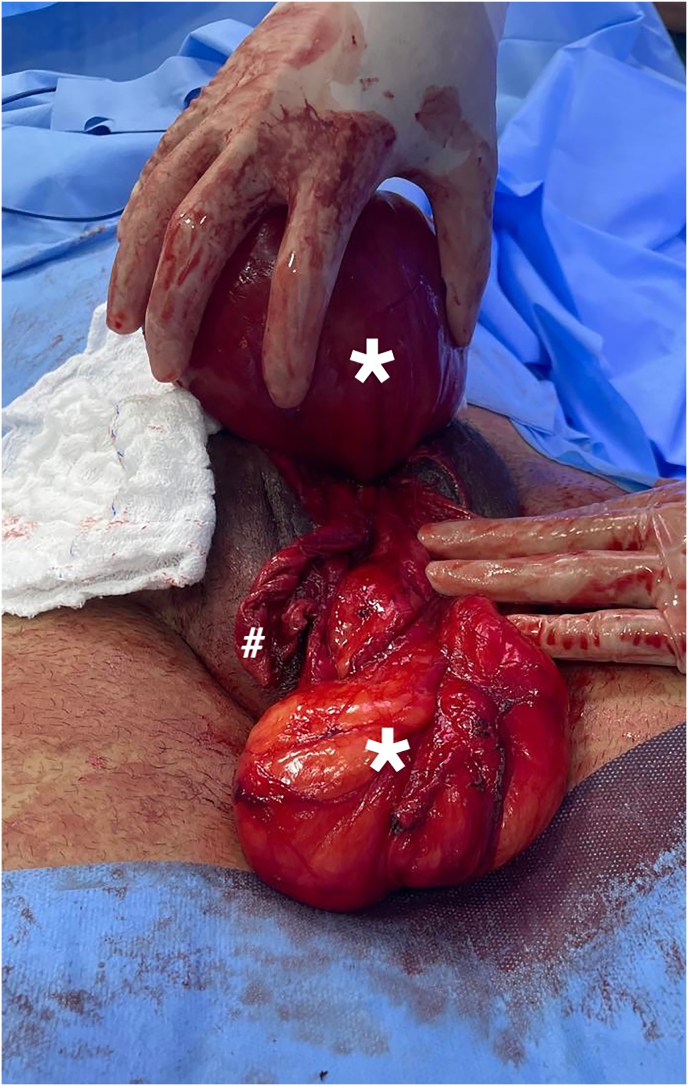
Intraoperative exposure of the surgical specimen. Legend: (*): liposarcoma; (#): left spermatic cord and testis.

Histopathological examination of the surgical specimen revealed a well-differentiated (low-grade) liposarcoma, with proliferation of mature adipocytes showing mild cellular atypia, absence of necrosis, and rare mitotic figures (02 mitoses/50 high-power fields). Circumferential surgical margins were free of neoplasia, and there was no evidence of lymphovascular or perineural invasion. Immunohistochemical analysis showed positivity for CDK4 and MDM2, confirming the diagnosis (Fig. 4).

**Fig. 4:**
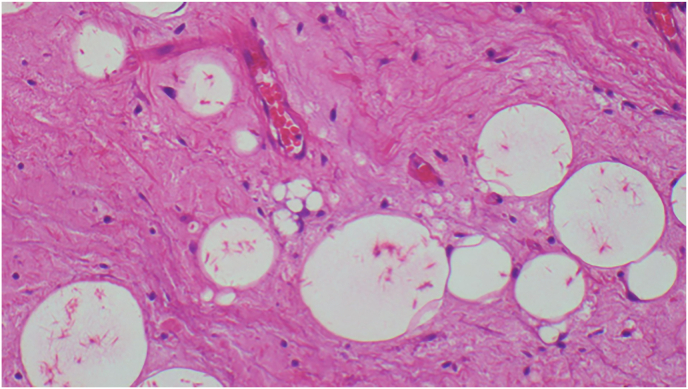
Histopathological findings showing adipocytic proliferation within a sclerotic stroma. A lipoblast is identified in the center of the field (hematoxylin and eosin stain, 100× magnification).

Clinical staging was defined as cT4N0Mx (Clinical Stage IB). No adjuvant therapy is planned. The patient had a successful postoperative course. He is currently asymptomatic and under outpatient follow-up with both the Urology and Clinical Oncology groups.

## 3 Discussion

Spermatic cord liposarcoma (LPS) is a rare malignant mesenchymal neoplasm, with an estimated 200 cases described in the worldwide literature, and may present in four distinct subtypes: well-differentiated (most frequent), myxoid, pleomorphic, and dedifferentiated.^1^, ^2^, ^3^ In a literature review by Fubial et al., the disease occurred between 24 and 79 years of age, with a mean of 61 years, and the most frequent clinical presentation was the slow growth of a painless mass above the testicles.^2^

Its main diagnostic challenge lies in its clinical similarity to other conditions, such as inguinal hernia, hydrocele, spermatocele, epididymitis, and lipoma, making preoperative diagnosis uncommon.^1^, ^2^, ^3^ In our patient's case, the history of prior hernioplasty acted as an additional confounding factor, directing clinical reasoning toward hernia recurrence and delaying suspicion of a neoplasm.

In this context, preoperative radiological assessment becomes essential for diagnostic clarification. Ultrasonography is generally the first imaging modality used in the evaluation of scrotal masses, but its findings in LPS may be nonspecific. They often consist of a heterogeneous mass, sometimes with central necrosis, as observed in this patient's ultrasound, but may be mistaken for a benign lipoma, especially in small lesions.^3^^,^^4^ Other methods may be used, such as computed tomography, but magnetic resonance imaging is considered the imaging modality of choice for the evaluation of soft tissue tumors, with greater accuracy in characterizing the lipomatous component, assessing lesion extent, and its relationship with adjacent structures, thereby aiding surgical planning^3^, ^4^, ^5^ Despite advances in imaging techniques, definitive differentiation between a benign lipoma and a well-differentiated LPS is virtually impossible based on radiological criteria alone, making histopathological confirmation indispensable.^5^

Regarding treatment, the gold standard is radical surgical resection, including orchiectomy with high ligation of the spermatic cord, aiming to achieve negative microscopic margins.^6^^,^^7^ The preferred surgical approach is the inguinal route, which allows primary vascular control of the spermatic cord prior to tumor manipulation.^7^ There is no evidence supporting the benefit of lymphadenectomy, as the well-differentiated histological subtype, the most frequent, is characterized by indolent clinical behavior and low potential for lymph node involvement and distant metastasis.^3^ Thus, the main risk of the disease is local recurrence, especially when the initial surgical resection is inadequate ^3^^,^^8^. The role of adjuvant therapy is not well established for low-grade tumors with negative margins.^3^ Emerging evidence supports adjuvant radiotherapy, particularly in cases with positive margins^1^^,^^2^ while chemotherapy is contraindicated due to the disease's resistance to this modality. Long-term follow-up is recommended due to the high risk of recurrence.^2^

## 4 Conclusion

Spermatic cord liposarcoma, although rare, should be considered a differential diagnosis in patients with inguinoscrotal masses. Preoperative diagnosis is uncommon in clinical practice, impacting prognosis after non-oncologic approaches, given that radical resection with negative margins is the treatment of choice. Adjuvant therapy, when indicated, should be performed with radiotherapy alone. Oncologic surveillance should be long-term due to the risk of local recurrence.

## CRediT authorship contribution statement

**Murilo Augusto Ferreira:** Writing – review & editing, Writing – original draft, Formal analysis, Conceptualization. **Gabriel Andrade Agareno:** Data curation, Conceptualization. **Fernando Aparecido Pazini:** Formal analysis. **Patrícia Mendes dos Santos Angolini:** Project administration. **Mateus Santos Matos:** Methodology. **Viktor Dias Magalhães:** Project administration. **Manuela Mascaro Dias:** Validation, Supervision, Formal analysis, Conceptualization. **Daniel Salerno Muzilli:** Supervision. **Thiago da Silveira Antoniassi:** Writing – review & editing, Supervision.
